# SupT1 Cell Infusion as a Possible Cell-Based Therapy for HIV: Results from a Pilot Study in Hu-PBMC BRGS Mice

**DOI:** 10.3390/vaccines4020013

**Published:** 2016-04-26

**Authors:** Jonathan Fior

**Affiliations:** Innovative Bioresearch, Milan 20154, Italy; jonathan.fior82@gmail.com; Tel.: +39-370-337-0489

**Keywords:** HIV, AIDS, cell-based therapy, humanized mice, SupT1 cells, CD4+ T cells

## Abstract

In a previous *in vitro* study, the SupT1 cell line was explored as a decoy target for HIV-1, proposing SupT1 cell infusion as a possible cell-based therapy for HIV. In the present work, the previous *in vitro* model was translated into an *in vivo* setting. Specifically, Hu-PBMC BRGS mice were infected with a high input of HIV-1 LAI (100,000 TCID_50_), and 40 million 30 Gy-irradiated SupT1 cells were infused weekly for 4 weeks as a therapy. Blood samples were taken to monitor CD4+ T cell count and viral load, and mice were monitored daily for signs of illness. At the earliest time point analyzed (Week 1), there was a significantly lower plasma viral load (~10-fold) in all animals treated with SupT1 cell infusion, associated with a higher CD4+ T cell count. At later time points, infection proceeded with robust viral replication and evident CD4+ T cell depletion, except in one mouse that showed complete suppression of viral replication and preservation of CD4+ T cell count. No morbidity or mortality was associated with SupT1 cell infusion. The interesting tendencies observed in the generated data suggest that this approach should be further investigated as a possible cell-based HIV therapy.

## 1. Introduction

In a previous work, the SupT1 cell line was explored as a decoy target for HIV-1 in an *in vitro* setting, proposing the infusion of SupT1 cells as a possible cell-based HIV therapy to prevent CD4+ T cell depletion as well as to render the virus less cytopathic [1]. In that study, it was observed that when HIV infection is performed in a SupT1/PBMC co-culture, the preferential infection of SupT1 cells can spare primary CD4+ T cells from infection and depletion. Accordingly, the rationale behind this possible approach is that moving infection toward the inoculated cells should prevent infection and depletion of the patient’s own CD4+ T cells and, therefore, AIDS. Furthermore, *in vitro* studies of HIV evolution showed that prolonged replication in SupT1 cells renders the virus less cytopathic and more sensitive to neutralization [2,3,4], indicating that *in vivo* virus replication in the infused SupT1 cells should also have a vaccination effect. Another interesting observation is that the HIV-1 Vif protein is essential for viral replication in primary CD4+ T cells but not in SupT1 cells [5]. Accordingly, pharmacologic inhibition of Vif could be combined with SupT1 cell infusion to further restrict viral replication to the inoculated SupT1 cells. Considering that APOBEC3G is expressed by different cell types, such as neuronal cells, astrocytes, and macrophages [6], pharmacologic inhibition of Vif may also have the benefit of acting on HIV reservoirs in the brain and other body areas. There are several molecules with promising anti-Vif activity [7,8,9]. Similarly, other HIV-1 accessory proteins that are not essential for replication in SupT1 cells (e.g., Vpr, Vpu, and Nef) [10] may also be the target of pharmacologic inhibition. Further considerations with regard to SupT1 cell infusion and its potential as an HIV therapy were made in a previous article [11]. The present pilot study aimed to translate the previously investigated *in vitro* model [1] into an *in vivo* setting. The study was performed in an *in vivo* model of HIV infection, generated with immunodeficient mice receiving an infusion of human PBMC (Hu-PBMC). Specifically, Hu-PBMC BRGS mice were infected with a high input of HIV-1 LAI followed by weekly SupT1 cell infusions as an HIV treatment over a 4-week study period. Longitudinal blood sample harvest over the 4-week treatment period was performed to monitor CD4+ T cell count and viral load, and mice were monitored daily for signs of illness. Positive and negative control groups were used to compare the results. At the earliest time point analyzed (Week 1), there was a significantly lower plasma viral load (~10-fold) in all mice treated with SupT1 cell infusion, associated with a higher CD4+ T cell count. At later time points, infection proceeded with robust viral replication and evident CD4+ T cell depletion, except in one mouse that showed complete suppression of viral replication (no virus detected anymore at Weeks 3 and 4) and preservation of CD4+ T cell count.

## 2. Materials and Methods

### 2.1. Mice

The mice used in the study were 18 unmanipulated male and female adult (aged 15–19 weeks at the time of treatment) BALB/c Rag2^tm1Fwa^ IL-2Rγc^tm1Cgn^ SIRPα^NOD^ (BRGS) mice [12]. BRGS mice are immunodeficient, devoid of murine T, B, and NK cells, and highly permissive to xenograft transplantation (SIRPα^NOD^ congenic). The animals were bred and kept in SOPF conditions in individually ventilated cages (up to seven mice per cage) of the ABSL3 facility of AXENIS (Paris, France). Sterile food and water were provided *ad libitum*. All animal experiments received approval from the local Animal Ethical Committee (CETEA 89, Institut Pasteur de Paris, France).

### 2.2. Cells

Hu-PBMC were isolated from a buffy coat using a Ficoll density gradient and kept frozen in liquid nitrogen in the presence of DMSO (10% final concentration) until further use. Cells were freshly thawed before inoculation into animals. The PBMC inoculum contained 60.5% human T cells, of which 46.8% were CD4+ T cells and 48.3% were CD8+ T cells (CD4:CD8 T cell ratio of 0.970). The T cell lymphoblastic lymphoma SupT1 cell line [13] was obtained from the DSMZ German Cell Line Collection (cell line number ACC140). The cells were cultured in RPMI-1640 Glutamax™ medium supplemented with 10% fetal bovine serum and 1% penicillin/streptomycin at 37 °C and in 5% CO_2_. SupT1 cells were injected into animals after 30 Gy irradiation delivered by an X-ray generator (X-Strahl).

### 2.3. HIV Virus

HIV-1 LAI (CXCR4-tropic) stock was obtained from Bertin Pharma (Montigny le Bretonneux, France) at a concentration of 418 × 10^3^ TCID_50_/mL (4.62 × 10^3^ pg/mL RT before clarification). Infection was performed intraperitoneally with 1 × 10^5^ TCID_50_, corresponding to 240 µL of the viral stock. One syringe was used per animal to reduce the risk of an injection accident.

### 2.4. Treatment and Control Groups

The 18 mice were divided into three groups (six mice per group): a treatment group (A), a positive control group (B), and a negative control group (C). The animals were ear-tagged with unique numbers. One week before onset of the study, the 18 selected mice were transferred to an ABSL3 isolator space (six cages, with three mice per cage). At the onset of the study, the six animals in treatment group A received 20 × 10^6^ Hu-PBMC intraperitoneally (T0), 40 × 10^6^ 30 Gy-irradiated SupT1 cells intraperitoneally at T + 2 h, and 1 × 10^5^ TCID_50_ of HIV-1 LAI virus intraperitoneally at T + 4 h [14]. Mice in group A also received three supplementary weekly intraperitoneal inoculations with 40 × 10^6^ 30 Gy-irradiated SupT1 cells each. The positive control group B did not receive SupT1 cells. The negative control group C did not receive SupT1 cells and was kept free of HIV infection. To avoid cross-contamination between the different groups, only animals belonging to the same group were placed together in cages (*i.e.*, six separate cages, each containing three animals of the same group). The three groups and injection schemes are summarized in Table 1.

### 2.5. Blood Sampling of the Animals

Blood samples (~50–100 µL) were harvested from the animals in EDTA-coated microtubes (Microvette® CB300 K2E) at the following time points: T + 1 week, T + 2 weeks, T + 3 weeks, and T + 4 weeks. In the case of treatment group A, blood sampling was performed before SupT1 cell inoculation. The blood samples were separated by centrifugation (10 min, 1000 ×*g*) into cell fractions and plasma fractions. The plasma fractions were kept frozen (−20°C) in microtubes until the end of the study. Blood cell leukocytes were isolated from the cell fraction on a Ficoll density gradient. Flow cytometric analysis of blood leukocytes was performed using a monoclonal antibody cocktail (Table 2) after incubation with human and murine FcR blocking reagents.

Incubations were performed in 96-well plates in the dark at 4 °C, and the following populations were successively gated: mouse leukocytes (hCD45−mCD45+), human leukocytes (hCD45+mCD45−), human T cells (hCD3+), and human T cell subsets based on CD4 *vs*. CD8 expression (CD4+CD8−, CD4−CD8+, and CD4+CD8+). Accordingly, SupT1 CD4+CD8+ T cells were distinguished from PBMC CD4+CD8− and CD4−CD8+ T cells by FACS analysis. However, this flow cytometry panel did not permit the distinction between SupT1 cells and PBMC-derived double-positive CD4+CD8+ T cells. All data acquisitions were performed with an LSR-II Fortessa flow cytometer interfaced with FACS-Diva software (BD Bioscience, San Jose, CA, USA). Data were analyzed using FlowJo 9.8 software (TreeStar Inc., Ashland, OR, USA), and graphs were plotted with GraphPad Prism-5 software (GraphPad Software Inc., La Jolla, CA, USA). Once all plasma samples were collected and stored, HIV viral load was measured by qPCR in a single run using the Generic HIV Charge Virale kit (BioCentric, Bandol, France). Viral load is expressed as HIV RNA copy number per mL of blood (detection threshold: 10^2^ HIV-1 RNA copies per mL).

### 2.6. Mouse Features

Each experimental condition included equivalent numbers of male and female mice, each harboring a unique ear tag number as its individual mouse ID (Table 3).

## 3. Results

**Figure 1:**
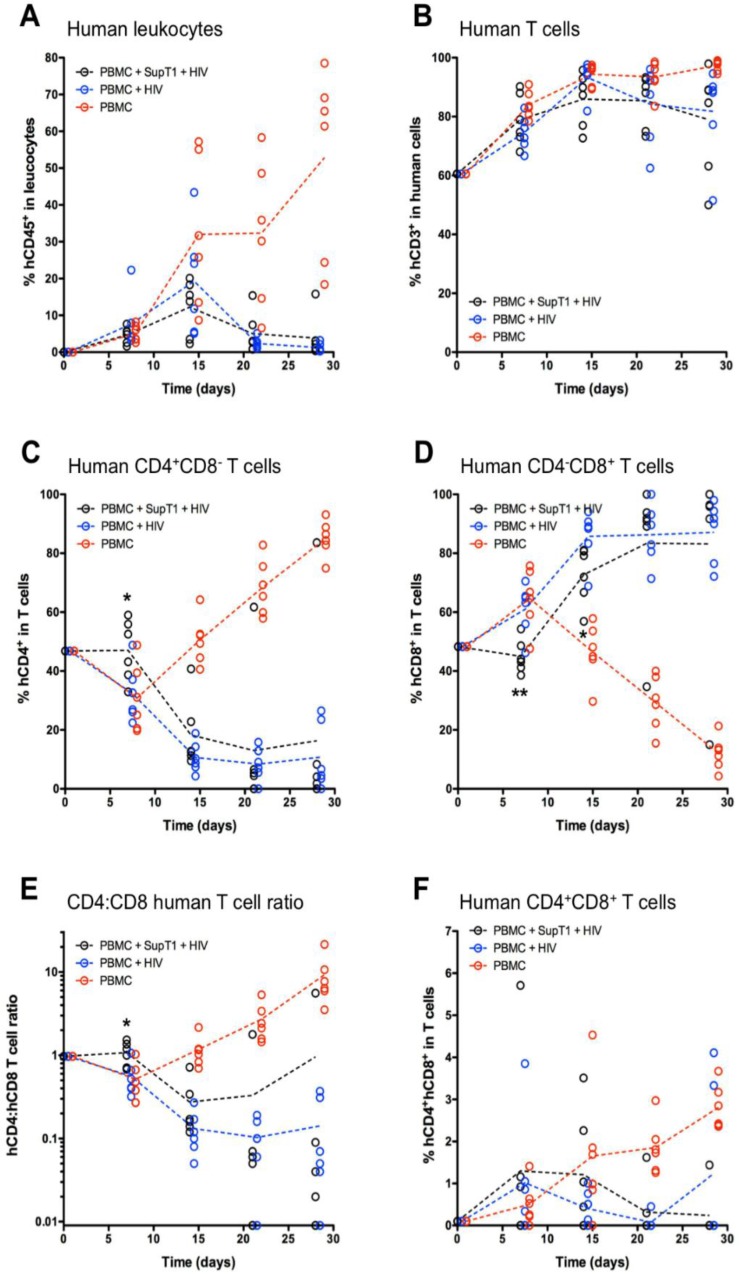
Monitoring of flow cytometry parameters. The graphs show individual values (open circles) and group mean values (dotted lines) for the following frequencies: (**A**) human leukocytes (hCD45+mCD45−) among total leukocytes; (**B**) T cells (hCD3+) among total human cells; (**C**) CD4+CD8− cells among total human T cells; (**D**) CD4−CD8+ cells among total human T cells; (**E**) the CD4:CD8 single-positive human T cell ratio; (**F**) CD4+CD8+ cells among total human T cells. For A, total leukocytes are intended as the sum of mouse (mCD45+) and human (hCD45+) leukocytes. For graphs in panels B, C, D, and F, the initial frequency in the inoculum is indicated at Day 0. * *p* < 0.05 and ** *p* < 0.01 (*t*-test between groups A and B).

The frequency of human leukocytes peaked at around 10%–20% of total leukocytes at Week 2 for both HIV-infected groups A (PBMC + SupT1 + HIV) and B (PBMC + HIV) and later decreased; by contrast, it showed a sustained increase over time in control group C (PBMC), to more than 50% of total leukocytes in the majority of animals (Figure 1A). In all groups, the vast majority of human cells belonged to the T cell lineage (Figure 1B). The frequency of CD4+CD8− cells among human T cells steadily increased in control group C after an initial drop at Week 1, whereas it decreased in HIV-infected groups A and B (Figure 1C). Logically, an opposite result was obtained for CD4−CD8+ T cells (Figure 1D), resulting in an increase (around 10-fold) in the CD4:CD8 single-positive T cell ratio in group C, and a decrease in groups A and B (Figure 1E). Statistical analysis revealed significant differences between groups A and B at Week 1 for the CD4+CD8− T cell frequency (*p* = 0.0289), CD4−CD8+ T cell frequency (*p* = 0.0034), and CD4:CD8 single-positive T cell ratio (*p* = 0.0163), as well as the CD4−CD8+ T cell frequency (*p* = 0.0363) at Week 2 (Figure 1C, D, E). Finally, the frequency of double-positive CD4+CD8+ T cells was similar in all groups, except at late time points (Weeks 3 and 4) in the negative control group, which showed increased levels (Figure 1F).

**Figure 2:**
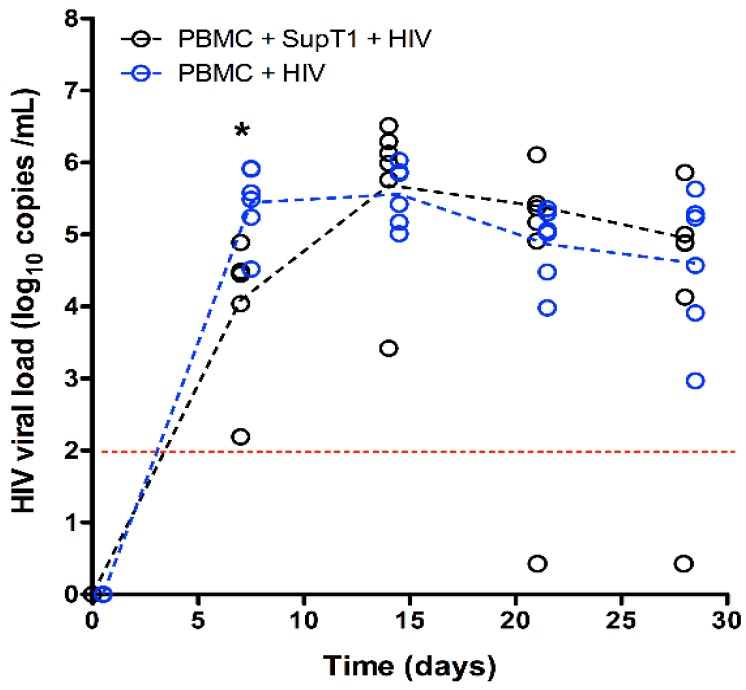
Measurement of HIV-1 viral load in humanized mouse blood. The plasma viral load is expressed as the number of copies per mL of plasma (on a base 10 log scale). The graph shows individual values (open circles) and group mean values (dotted lines). The red dotted line shows the detection threshold of the assay. Individual values under the detection threshold were excluded from calculation of the mean value and from statistical analysis (*i.e.*, calculations are for animals with a detectable viral load only). * *p* < 0.05 (*t*-test between groups A and B).

The HIV-1 viral load was quantified by qPCR in plasma samples collected from the animals over time (Figure 2). Virus was not detected in samples from negative control group C. The viral load in both HIV-infected groups A and B was similar over time, except at Week 1, when it was ~10-fold lower in the animals of group A (*p* = 0.0127) (Figure 2). This significantly lower plasma viral load seems to correlate with the higher CD4+ T cell levels of group A measured at Week 1. Of note, one individual (mouse 50) in group A exhibited complete suppression of viral replication (no virus detected anymore at Weeks 3 and 4) and preservation of CD4+ T cell count. This mouse behaved similarly to animals in negative control group C, with maintenance of detectable levels of human leukocytes in blood and increased levels of CD4+ T cells over time.

**Figure 3:**
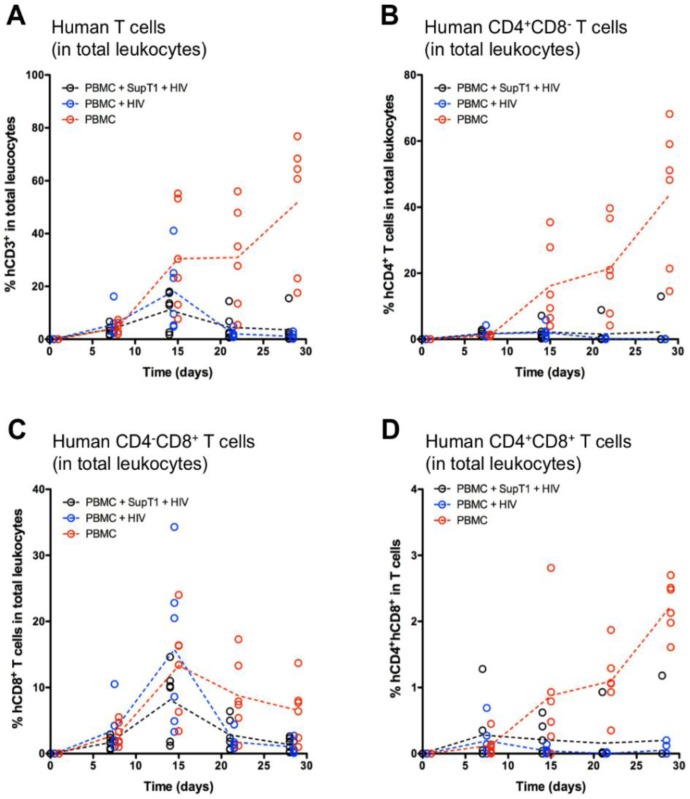
Normalized parameters in total leukocytes. The graphs show individual values (open circles) and group mean values (dotted lines) for the following frequencies: (**A**) human T cells (hCD45+mCD45−hCD3+) among total leukocytes; (**B**) human CD4+CD8− T cells among total leukocytes; (**C**) human CD4−CD8+ T cells among total leukocytes; (**D**) human CD4+CD8+ T cells among total leukocytes. Total leukocytes are intended as the sum of mouse (mCD45+) and human (hCD45+) leukocytes.

Because the frequency of human cells was strikingly different between the HIV-infected groups A/B and the negative control group C, the relative frequency of human T cells, CD4+CD8− T cells, CD4−CD8+ T cells, and CD4+CD8+ T cells among total leukocytes was also calculated. This normalization step provided a better understanding of the relative kinetics for each of these cell populations. It was clear that the human T cell frequency increased in all groups until Week 2, and then dropped to low levels in the HIV-infected groups (Figure 3A). Interestingly, human CD4+CD8− T cell expansion was only observed in the negative control group from Week 2 (Figure 3B), whereas human CD4−CD8+ T cell expansion kinetics appeared to be similar in all groups, with better maintenance in the negative control group at Weeks 3 and 4 (Figure 3C). Only negative control group C showed accumulation of double-positive CD4+CD8+ T cells over time (Figure 3D). Once again, mouse 50 was noticeable in group A, and statistical analysis did not reveal any significant difference in these parameters between groups A and B.

These findings indicate that SupT1 cells accounted for a small fraction of the total CD4+CD8+ T cell population, which in turn indicates that, despite the high doses of SupT1 cells infused, irradiating the cells prior to inoculation efficiently prevented SupT1 cell replication. It is therefore possible to conclude that, while mouse 50 showed accumulation of CD4+CD8+ T cells at late time points, the majority of these double positive T cells are likely represented by PBMC-derived cells and not SupT1 cells. The mice receiving SupT1 cell infusion were monitored regularly for signs of illness, for instance as a consequence of graft-*versus*-host disease progression. In this regard, all mice in group A successfully survived the treatment and no evidence of morbidity associated with the weekly SupT1 cell infusions was observed. The individual numerical data for each mouse and the individual FACS dot plots from which the data were extracted are presented, respectively, in Table S1 and Figure S1.

## 4. Discussion

In summary, effective human cell intraperitoneal transplantation and subsequent HIV-1 LAI intraperitoneal infection were observed in all BRGS mice in groups A and B. HIV-1 infection in this experimental model leads to a decrease in human cells over time, especially human CD4+ T cells, thereby reproducing the dynamics of CD4+ T cell depletion observed in human HIV infection. As an HIV treatment, SupT1 cell infusion had an impact on viral replication and CD4+ T cell count at the earliest time point analyzed (Week 1), with one mouse eventually behaving like an uninfected mouse. Because some interesting tendencies were observed in the generated data, such as lower viral replication and potentially preserved CD4+ T cell frequency at Week 1, a larger sample size as well as down-titration of the virus dose may reveal clearer differences between treatment and control groups in future studies. In particular, future studies may investigate the conditions that caused the interesting result observed in mouse 50, which exhibited a sustained decrease in HIV replication and CD4+ T cell depletion, a result that would be the goal of an effective HIV treatment. Specifically, it would be necessary to determine whether this phenomenon is a consequence of SupT1 cell infusion or is due to other factors, for instance a less efficient initial infection in this individual. Although at this stage it is not possible to determine it, some considerations can be made using the available data. A first consideration is that all mice were engrafted with PBMC obtained from a single donor, excluding donor variability as a possible cause. A second consideration is that this sustained inhibitory effect on viral replication was observed in an animal of treatment group A, whereas no animal in positive control group B behaved similarly. Considering the interesting tendency to have a significantly lower viral replication at Week 1 in animals treated with SupT1 cell infusion, the sustained inhibition seems to be a phenomenon related to the presence of SupT1 cells. It is therefore possible that the combination of a very aggressive viral strain such as HIV-1 LAI and a relatively high viral input hides the potential efficacy of the treatment, resulting in only one animal showing the desired outcome. Accordingly, along with a lower virus dose, using less aggressive HIV strains may also yield better results in future studies. Another consideration is that, while supporting productive HIV-1 infection and viral DNA integration, irradiated and therefore growth-arrested SupT1 cells may produce less virus than actively dividing cells, which could correlate with the inhibitory effect on viral replication. Analysis of genetic diversity and attenuation of these circulating viral populations may also be interesting. With regard to treatment safety, in this study safe *in vivo* inoculation with high doses of irradiated SupT1 cells was possible in a highly immunodeficient mouse strain such as BRGS, indicating safety for the treatment of immunocompromised hosts such as AIDS patients. Most notably, presence of SupT1 cells was almost undetectable at late time points, indicating that SupT1 cell infusion had no tumorigenic consequences.

## 5. Conclusions

In conclusion, the interesting results suggest that this approach deserves further investigation as a possible cell-based therapy for HIV.

## Figures and Tables

**Table 1:** Injection schemes.

Group	n	T0	T + 2 hours	T + 4 hours	T + 1 week	T + 2 weeks	T + 3 weeks
A	6	20 × 10^6^ PBMC	40 × 10^6^ SupT1	10^5^ TCID_50_ HIV LAI	40 × 10^6^ SupT1	40 × 10^6^ SupT1	40 × 10^6^ SupT1
B (pos-CT)	6	20 × 10^6^ PBMC	-	10^5^ TCID_50_ HIV LAI	-	-	-
C (neg-CT)	6	20 × 10^6^ PBMC	-	-	-	-	-

pos-CT: positive control; neg-CT: negative control. The animals were maintained in the study for a total of 4 weeks and monitored regularly for signs of illness, for instance as a consequence of graft-*versus*-host disease progression.

**Table 2:** Flow cytometry reagents.

Target	Label	Clone	Origin	Dilution
hCD45	PerCP	H130	BioLegend	1:50
mCD45	APC E780	30-F11	eBioscience	1:200
hCD3	E450	UCHT1	eBioscience	1:50
hCD4	PE	MEM-241	ImmunoTools	1:25
hCD8	FITC	MEM-31	ImmunoTools	1:12.5

**Table 3:** Individual mouse characteristics.

Group	A	B	C
Content	PBMC/SupT1/HIV	PBMC/HIV	PBMC
HIV-1 Status	+	+	−
# mice	6	6	6
Mouse ID (age at onset)	F 40 (17 weeks)	F 43 (19 weeks)	F 46 (19 weeks)
	F 41 (17 weeks)	F 44 (19 weeks)	F 47 (19 weeks)
	F 42 (17 weeks)	F 45 (19 weeks)	F 48 (19 weeks)
	M 49 (15 weeks)	M 52 (15 weeks)	M 55 (15 weeks)
	M 50 (15 weeks)	M 53 (15 weeks)	M 56 (15 weeks)
	M 51 (15 weeks)	M 54 (15 weeks)	M 57 (15 weeks)

All males were 15 weeks old, and females ranged between 17 and 19 weeks of age at the time of cell injection and/or HIV-1 inoculation. F: female; M: male; weeks: weeks of age.

## Supplementary Materials

The following are available online at www.mdpi.com/2076-393X/4/2/13/s1, Figure S1: Individual FACS dot plots, Table S1: Individual numerical data for each mouse.
