# Supplementary material for: SupT1 Cell Infusion as a Possible Cell-Based Therapy for HIV: Results from a Pilot Study in Hu-PBMC BRGS Mice
Source: Vaccines (Basel). 2016 Apr 26;4(2):13. doi: 10.3390/vaccines4020013 (PMC4931630; doi:10.3390/vaccines4020013)
Supplement: Supplementary file 1 [file vaccines-04-00013-s001.zip › vaccines-120074-Figure S1.pdf]

FACS dot plots

TIME: +1 WEEK

MOUSE 40

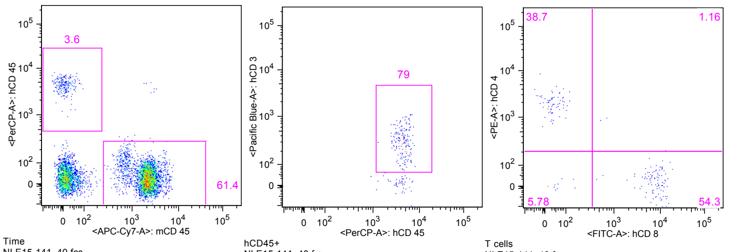

Time
NLE15-141_40.fcs
Event Count: 6087

hCD45+
NLE15-141_40.fcs
Event Count: 219

T cells
NLE15-141_40.fcs
Event Count: 173

MOUSE 41

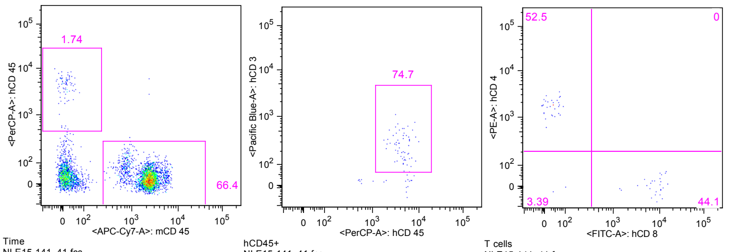

Time
NLE15-141_41.fcs
Event Count: 4539

hCD45+
NLE15-141_41.fcs
Event Count: 79

T cells
NLE15-141_41.fcs
Event Count: 59

MOUSE 42

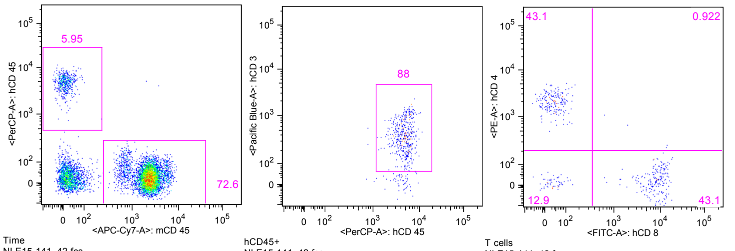

Time
NLE15-141_42.fcs
Event Count: 8286

hCD45+
NLE15-141_42.fcs
Event Count: 493

T cells
NLE15-141_42.fcs
Event Count: 434

MOUSE 43

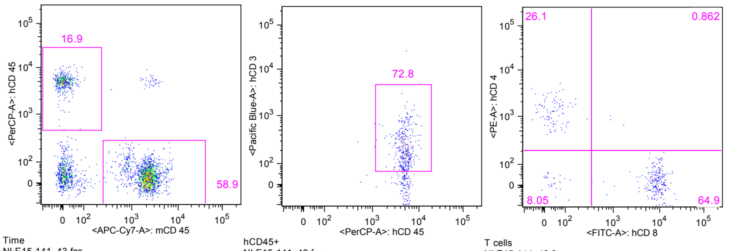

Time
NLE15-141_43.fcs
Event Count: 2831

hCD45+
NLE15-141_43.fcs
Event Count: 478

T cells
NLE15-141_43.fcs
Event Count: 348

MOUSE 44

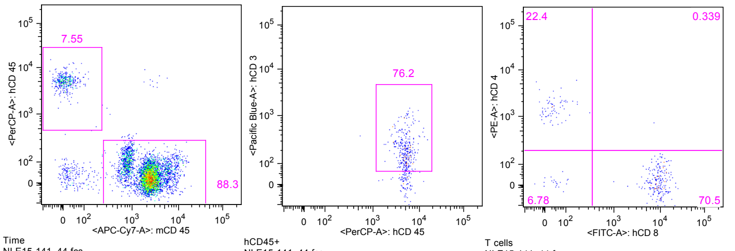

Time
NLE15-141_44.fcs
Event Count: 5129

hCD45+
NLE15-141_44.fcs
Event Count: 387

T cells
NLE15-141_44.fcs
Event Count: 295

MOUSE 45

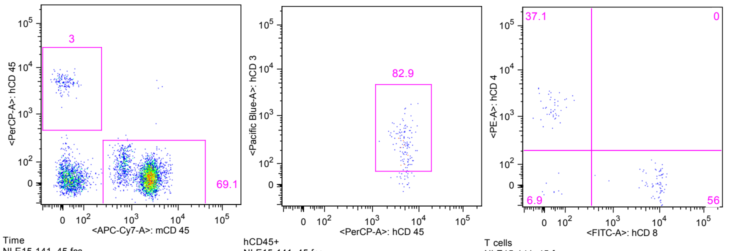

Time
NLE15-141_45.fcs
Event Count: 4669

hCD45+
NLE15-141_45.fcs
Event Count: 140

T cells
NLE15-141_45.fcs
Event Count: 116

MOUSE 46

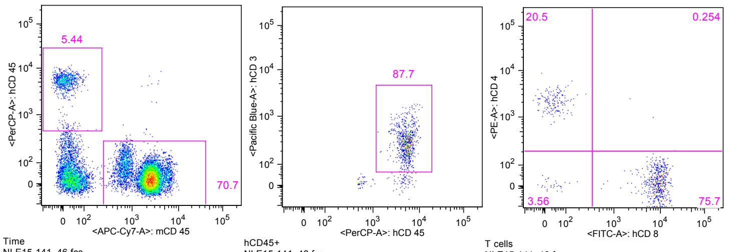

Time
NLE15-141_46.fcs
Event Count: 16475

hCD45+
NLE15-141_46.fcs
Event Count: 896

T cells
NLE15-141_46.fcs
Event Count: 786

MOUSE 47

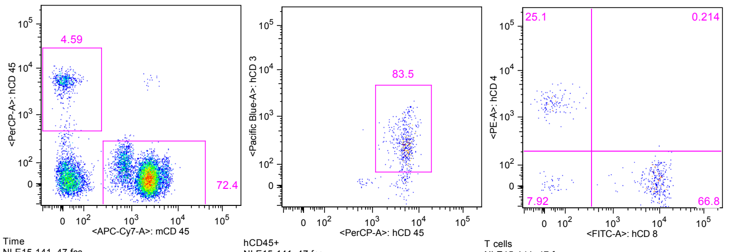

Time
NLE15-141_47.fcs
Event Count: 12186

hCD45+
NLE15-141_47.fcs
Event Count: 559

T cells
NLE15-141_47.fcs
Event Count: 467

MOUSE 48

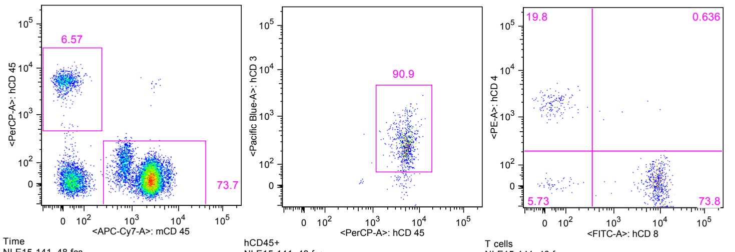

Time
NLE15-141_48.fcs
Event Count: 13166

hCD45+
NLE15-141_48.fcs
Event Count: 865

T cells
NLE15-141_48.fcs
Event Count: 786

MOUSE 49

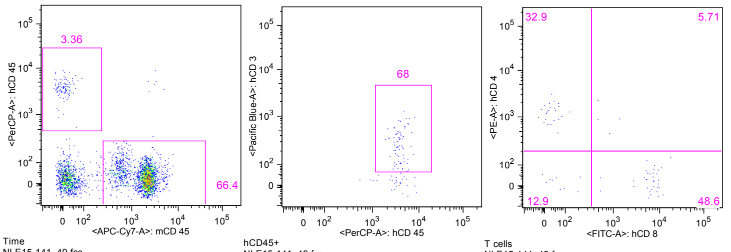

Time
NLE15-141_49.fcs
Event Count: 3061

hCD45+
NLE15-141_49.fcs
Event Count: 103

T cells
NLE15-141_49.fcs
Event Count: 70

MOUSE 50

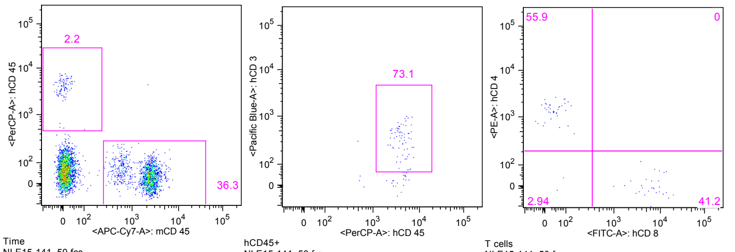

Time
NLE15-141_50.fcs
Event Count: 4221

hCD45+
NLE15-141_50.fcs
Event Count: 93

T cells
NLE15-141_50.fcs
Event Count: 68

MOUSE 51

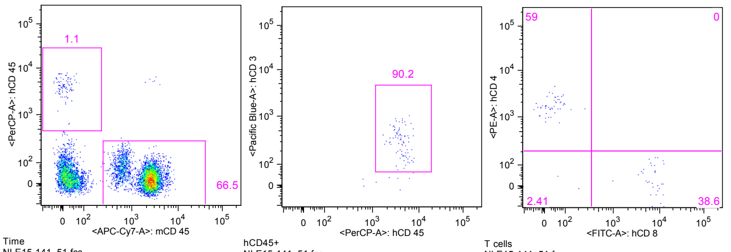

Time
NLE15-141_51.fcs
Event Count: 8393

hCD45+
NLE15-141_51.fcs
Event Count: 92

T cells
NLE15-141_51.fcs
Event Count: 83

FACS dot plots

TIME: +1 WEEK

MOUSE 52

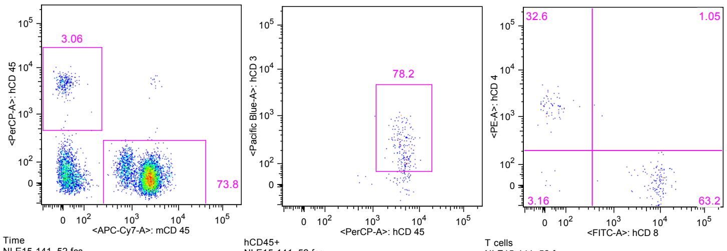

Time
NLE15-141_52.fcs
Event Count: 7929

hCD45+
NLE15-141_52.fcs
Event Count: 243

T cells
NLE15-141_52.fcs
Event Count: 190

MOUSE 53

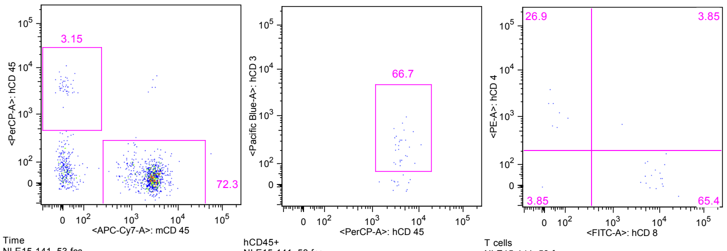

Time
NLE15-141_53.fcs
Event Count: 1239

hCD45+
NLE15-141_53.fcs
Event Count: 39

T cells
NLE15-141_53.fcs
Event Count: 26

MOUSE 54

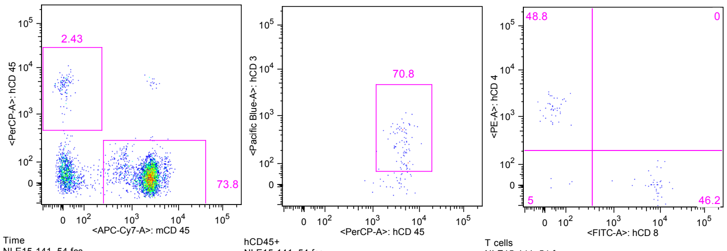

Time
NLE15-141_54.fcs
Event Count: 4644

hCD45+
NLE15-141_54.fcs
Event Count: 113

T cells
NLE15-141_54.fcs
Event Count: 80

MOUSE 55

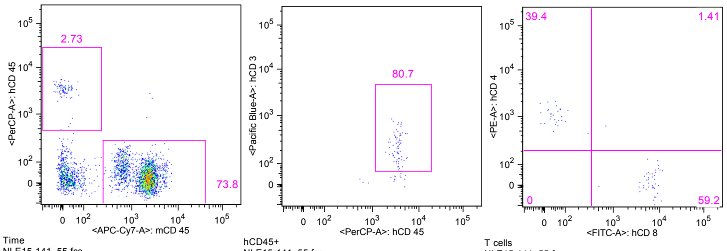

Time
NLE15-141_55.fcs
Event Count: 3221

hCD45+
NLE15-141_55.fcs
Event Count: 88

T cells
NLE15-141_55.fcs
Event Count: 71

MOUSE 56

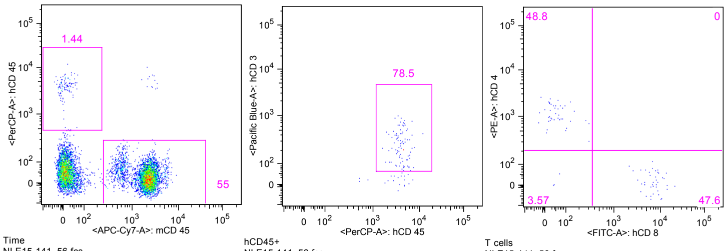

Time
NLE15-141_56.fcs
Event Count: 7453

hCD45+
NLE15-141_56.fcs
Event Count: 107

T cells
NLE15-141_56.fcs
Event Count: 84

MOUSE 57

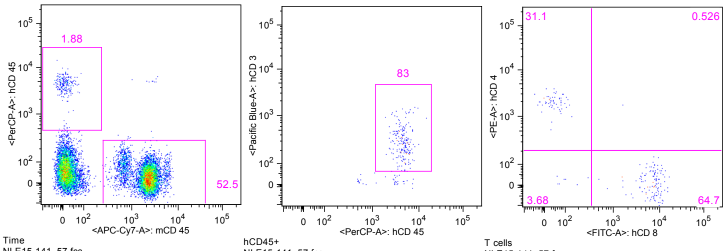

Time
NLE15-141_57.fcs
Event Count: 12178

hCD45+
NLE15-141_57.fcs
Event Count: 229

T cells
NLE15-141_57.fcs
Event Count: 190

FACS dot plots TIME: +2 WEEKS

MOUSE 40

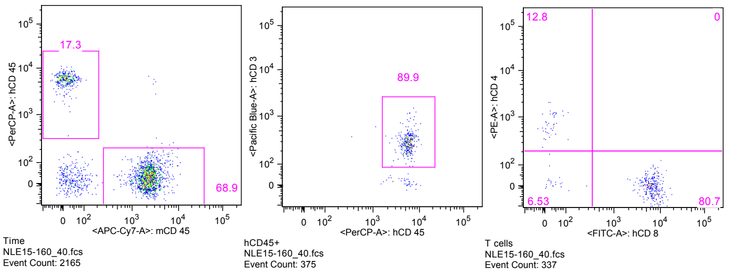

MOUSE 41

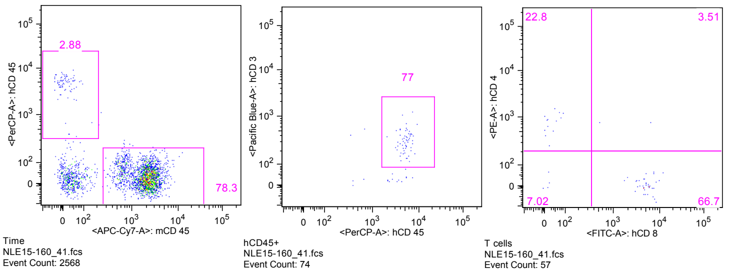

MOUSE 42

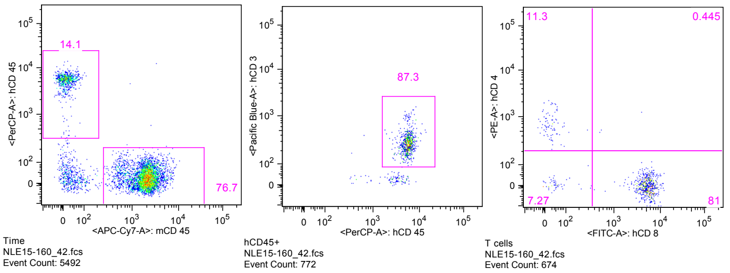

MOUSE 43

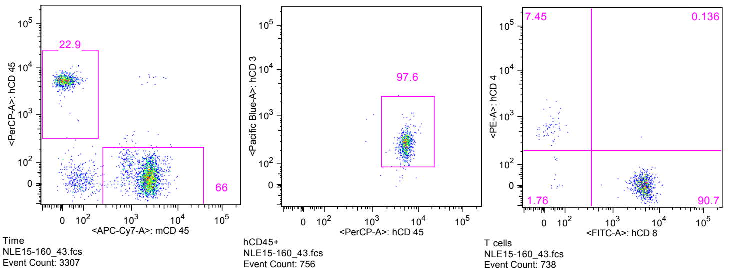

MOUSE 44

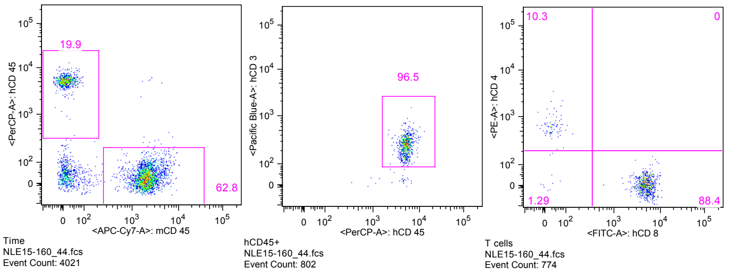

MOUSE 45

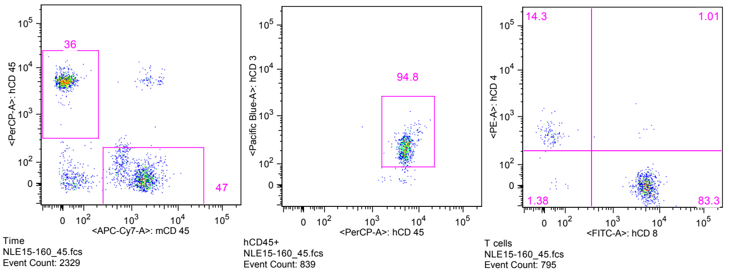

MOUSE 46

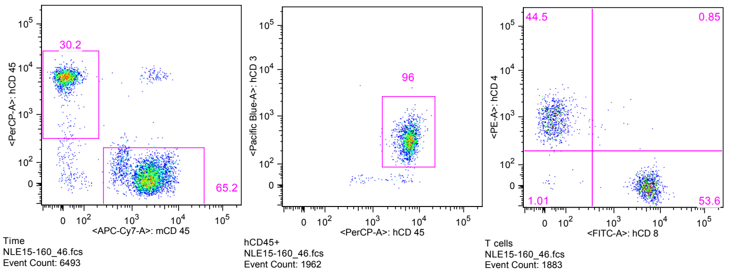

MOUSE 47

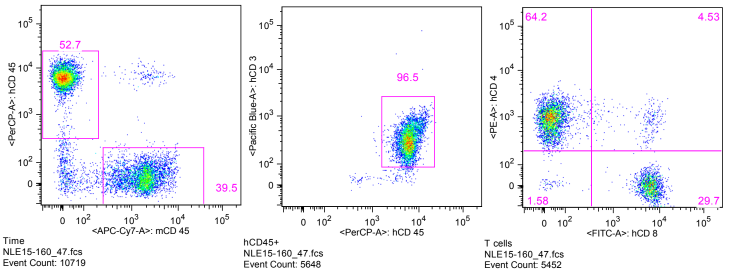

MOUSE 48

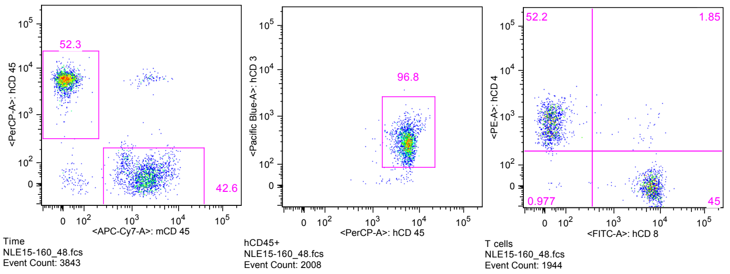

MOUSE 49

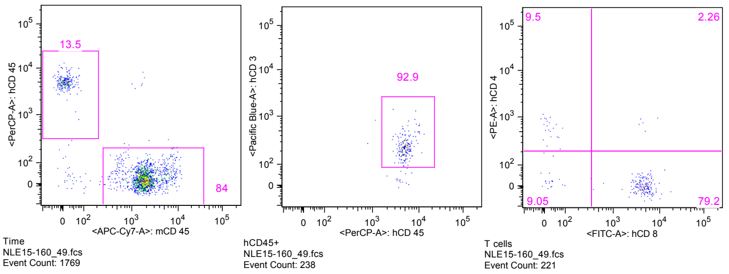

MOUSE 50

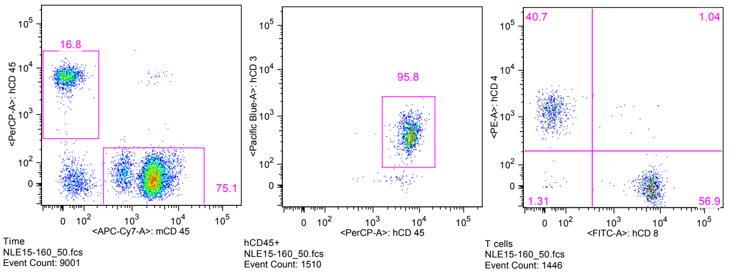

MOUSE 51

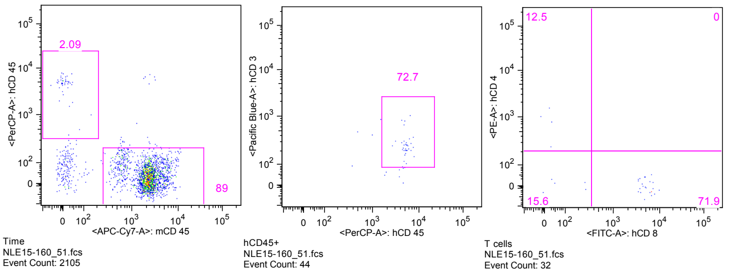

FACS dot plots

TIME: +2 WEEKS

MOUSE 52

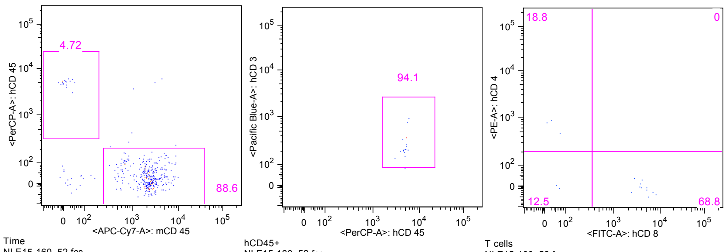

Time
NLE15-160_52.fcs
Event Count: 360

hCD45+
NLE15-160_52.fcs
Event Count: 17

T cells
NLE15-160_52.fcs
Event Count: 16

MOUSE 53

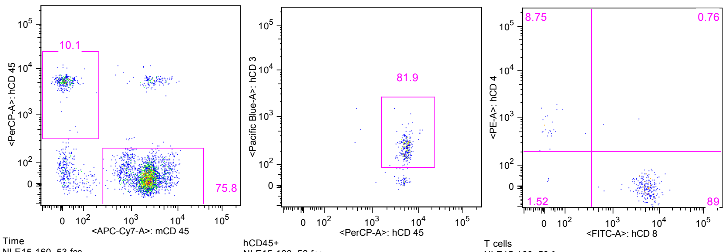

Time
NLE15-160_53.fcs
Event Count: 3164

hCD45+
NLE15-160_53.fcs
Event Count: 321

T cells
NLE15-160_53.fcs
Event Count: 263

MOUSE 54

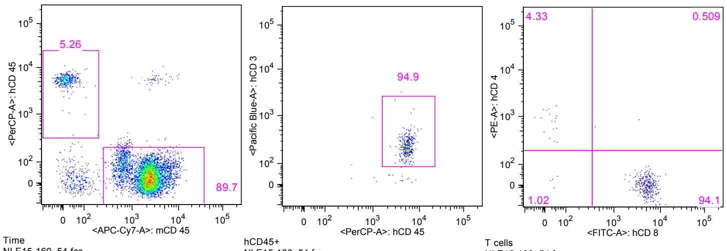

Time
NLE15-160_54.fcs
Event Count: 7875

hCD45+
NLE15-160_54.fcs
Event Count: 414

T cells
NLE15-160_54.fcs
Event Count: 393

MOUSE 55

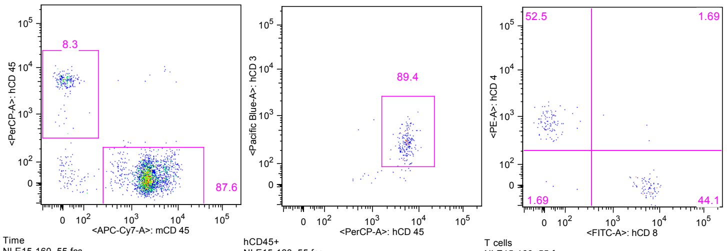

Time
NLE15-160_55.fcs
Event Count: 2385

hCD45+
NLE15-160_55.fcs
Event Count: 198

T cells
NLE15-160_55.fcs
Event Count: 177

MOUSE 56

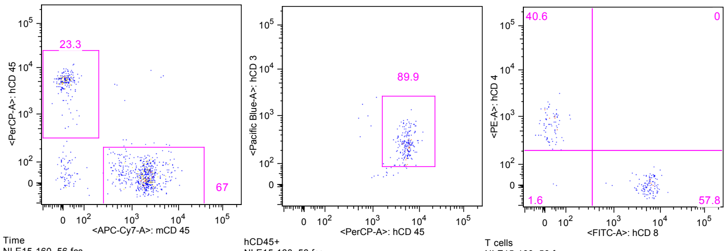

Time
NLE15-160_56.fcs
Event Count: 893

hCD45+
NLE15-160_56.fcs
Event Count: 208

T cells
NLE15-160_56.fcs
Event Count: 187

MOUSE 57

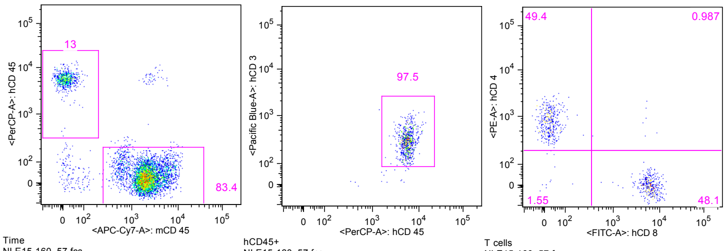

Time
NLE15-160_57.fcs
Event Count: 5585

hCD45+
NLE15-160_57.fcs
Event Count: 727

T cells
NLE15-160_57.fcs
Event Count: 709

FACS dot plots

TIME: +3 WEEKS

MOUSE 40

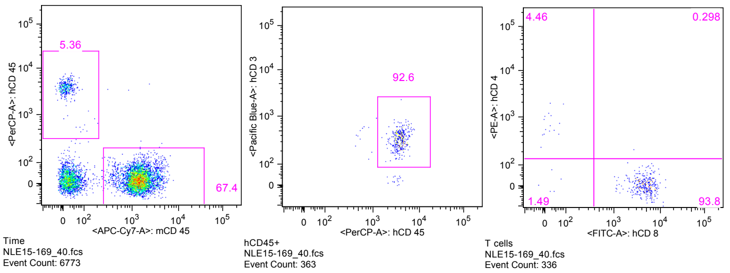

MOUSE 41

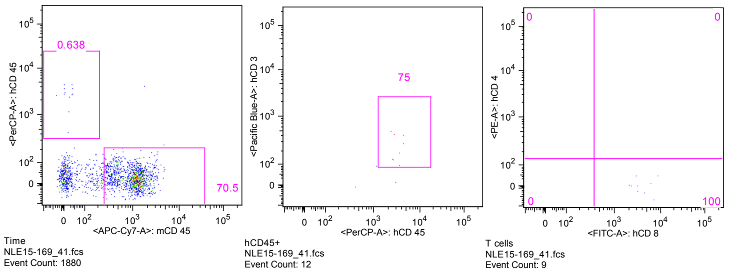

MOUSE 42

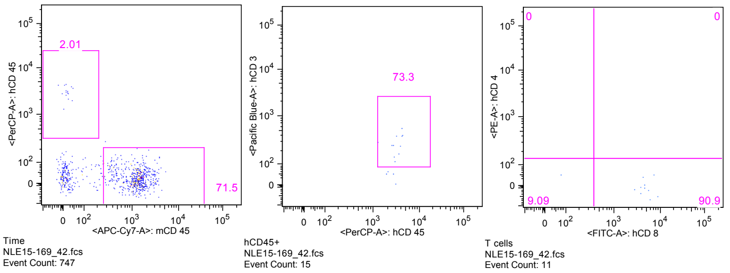

MOUSE 43

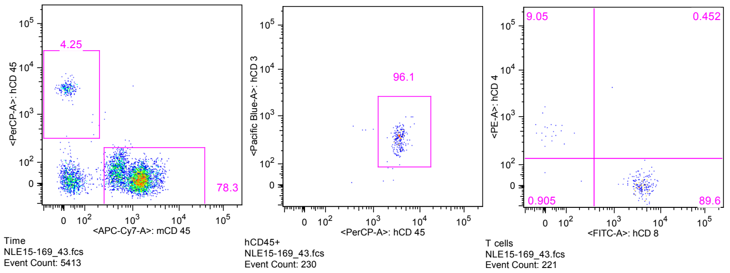

MOUSE 44

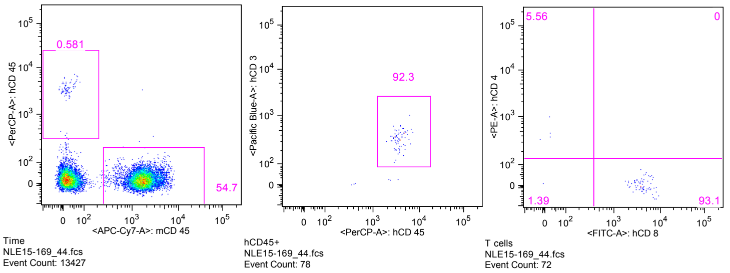

MOUSE 45

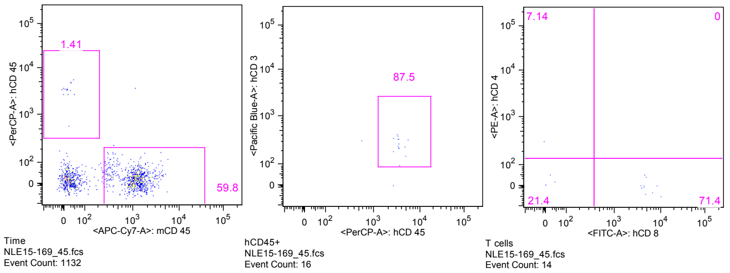

MOUSE 46

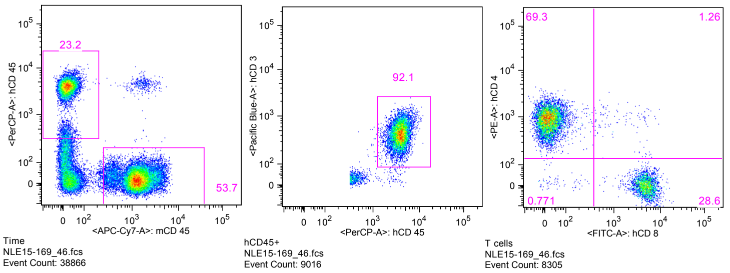

MOUSE 47

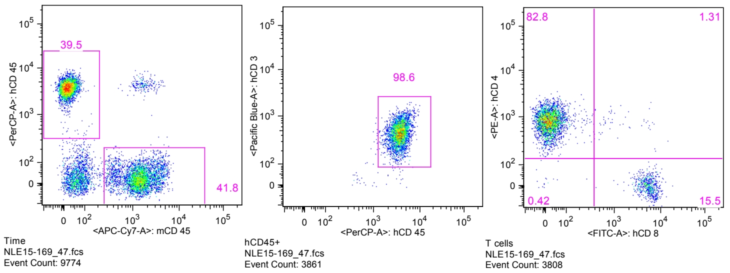

MOUSE 48

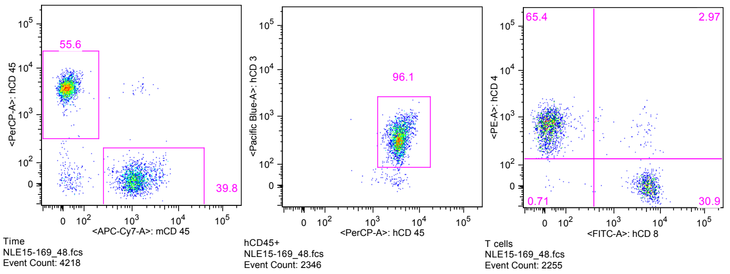

MOUSE 49

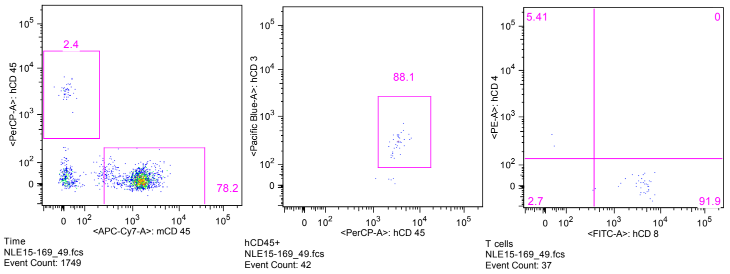

MOUSE 50

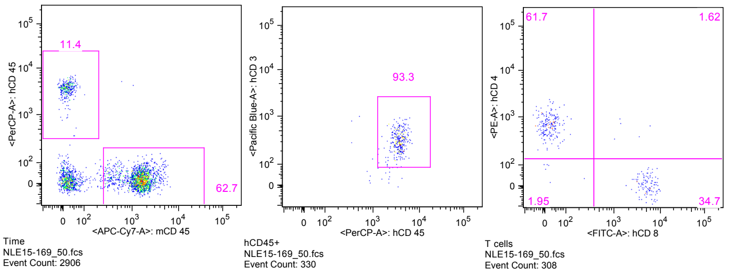

MOUSE 51

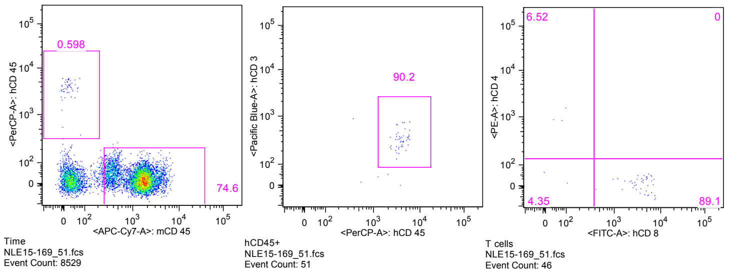

FACS dot plots

TIME: +3 WEEKS

MOUSE 52

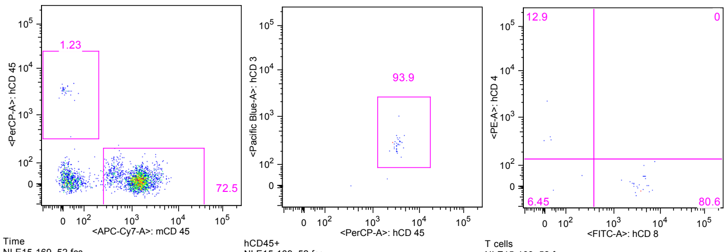

Time
NLE15-169_52.fcs
Event Count: 2678

hCD45+
NLE15-169_52.fcs
Event Count: 33

T cells
NLE15-169_52.fcs
Event Count: 31

MOUSE 53

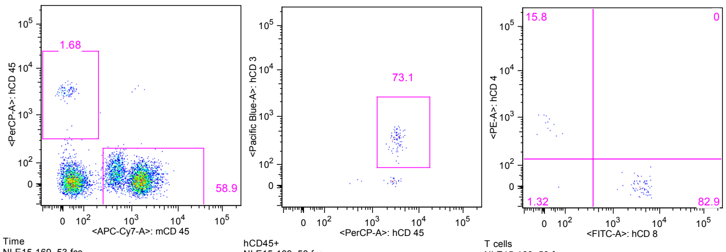

Time
NLE15-169_53.fcs
Event Count: 6208

hCD45+
NLE15-169_53.fcs
Event Count: 104

T cells
NLE15-169_53.fcs
Event Count: 76

MOUSE 54

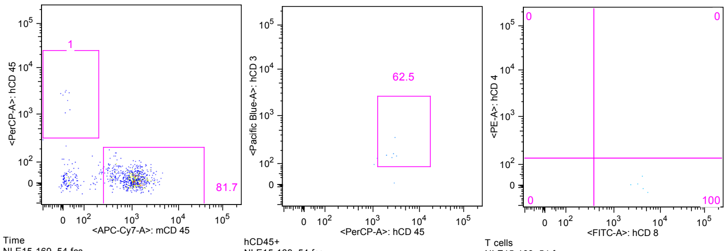

Time
NLE15-169_54.fcs
Event Count: 798

hCD45+
NLE15-169_54.fcs
Event Count: 8

T cells
NLE15-169_54.fcs
Event Count: 5

MOUSE 55

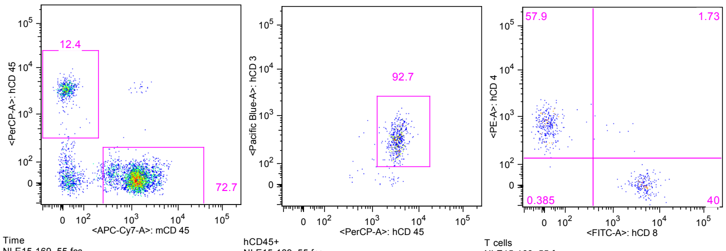

Time
NLE15-169_55.fcs
Event Count: 4523

hCD45+
NLE15-169_55.fcs
Event Count: 561

T cells
NLE15-169_55.fcs
Event Count: 520

MOUSE 56

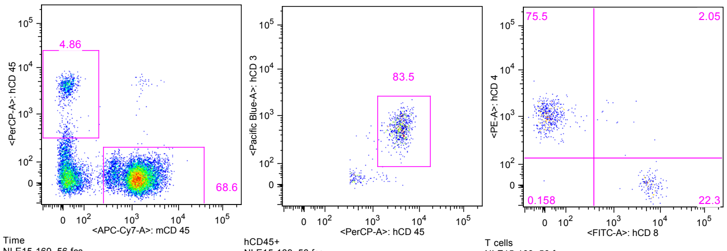

Time
NLE15-169_56.fcs
Event Count: 15595

hCD45+
NLE15-169_56.fcs
Event Count: 758

T cells
NLE15-169_56.fcs
Event Count: 633

MOUSE 57

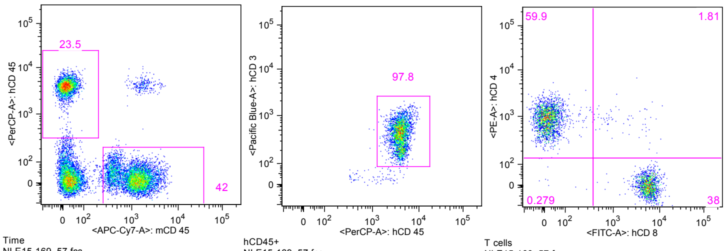

Time
NLE15-169_57.fcs
Event Count: 15625

hCD45+
NLE15-169_57.fcs
Event Count: 3666

T cells
NLE15-169_57.fcs
Event Count: 3584

FACS dot plots

TIME: +4 WEEKS

MOUSE 40

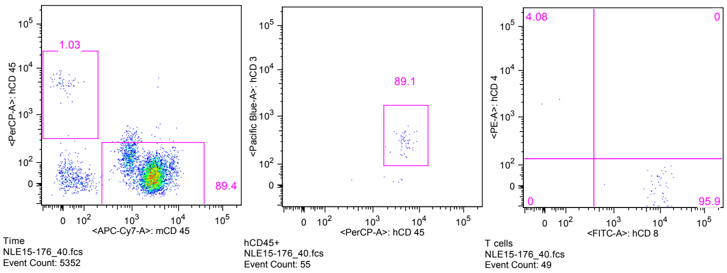

MOUSE 41

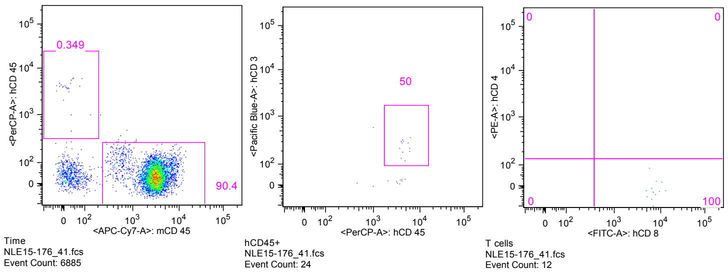

MOUSE 42

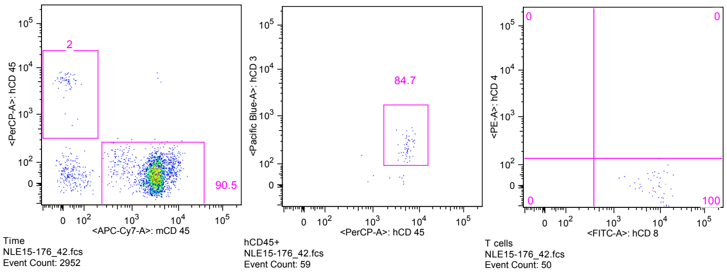

MOUSE 43

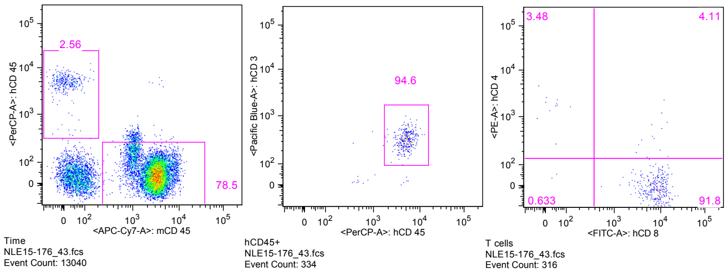

MOUSE 44

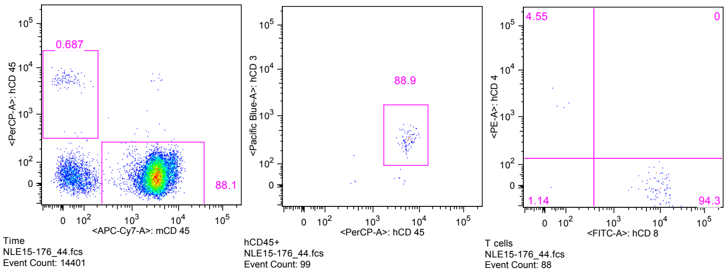

MOUSE 45

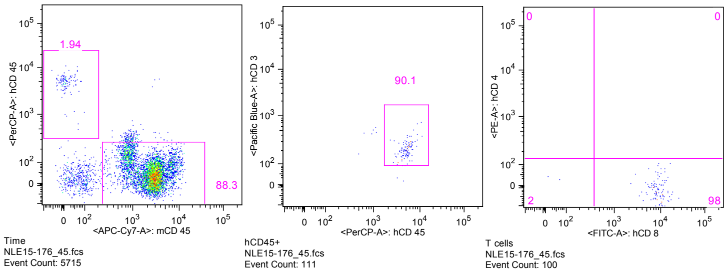

MOUSE 46

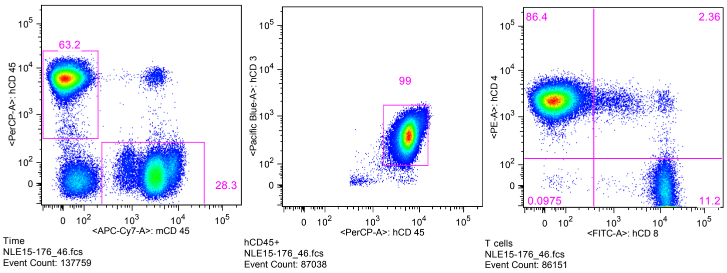

MOUSE 47

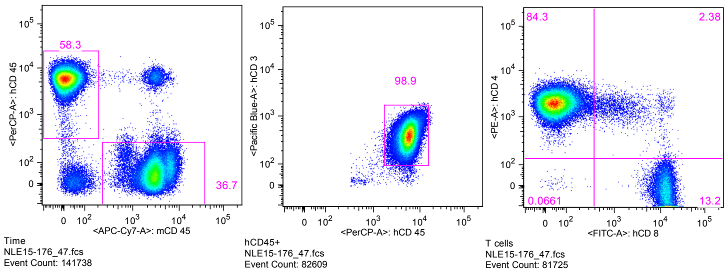

MOUSE 48

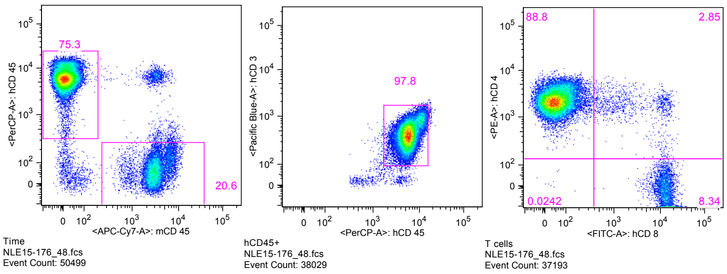

MOUSE 49

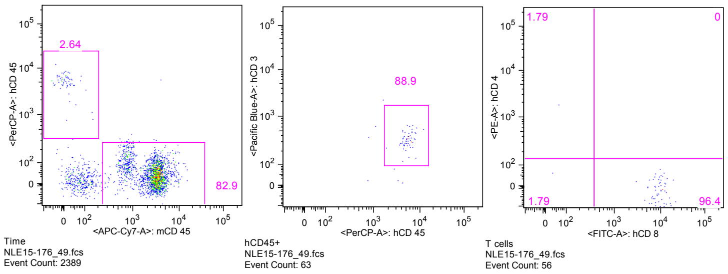

MOUSE 50

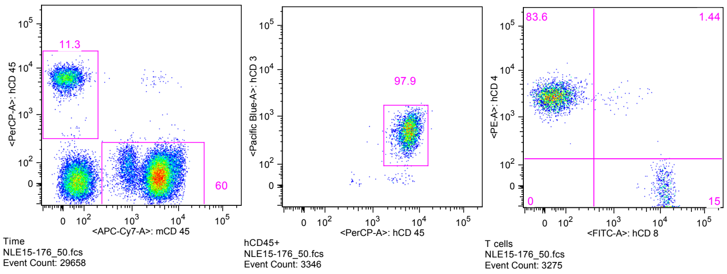

MOUSE 51

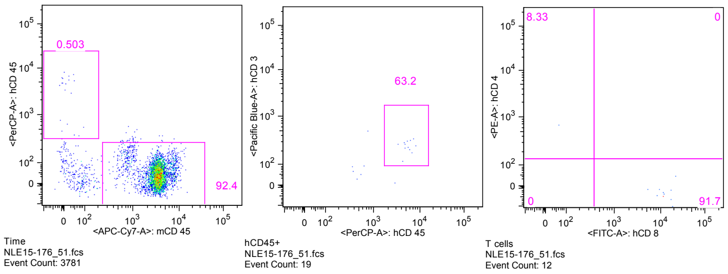

FACS dot plots

TIME: +4 WEEKS

MOUSE 52

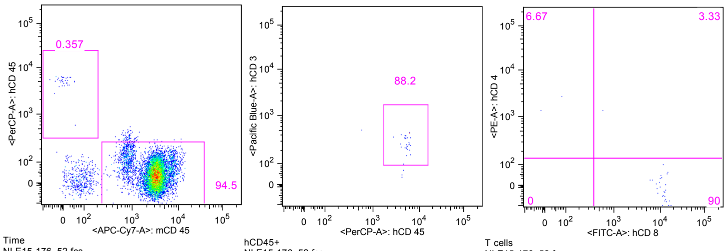

Time
NLE15-176_52.fcs
Event Count: 9521

hCD45+
NLE15-176_52.fcs
Event Count: 34

T cells
NLE15-176_52.fcs
Event Count: 30

MOUSE 53

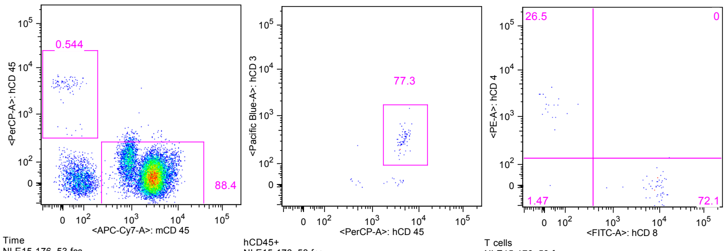

Time
NLE15-176_53.fcs
Event Count: 16185

hCD45+
NLE15-176_53.fcs
Event Count: 88

T cells
NLE15-176_53.fcs
Event Count: 68

MOUSE 54

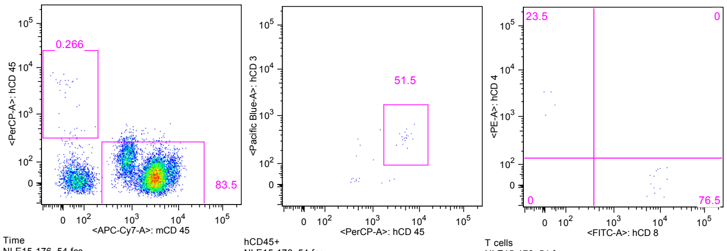

Time
NLE15-176_54.fcs
Event Count: 12391

hCD45+
NLE15-176_54.fcs
Event Count: 33

T cells
NLE15-176_54.fcs
Event Count: 17

MOUSE 55

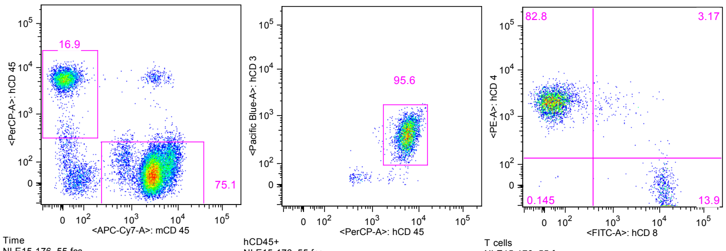

Time
NLE15-176_55.fcs
Event Count: 21360

hCD45+
NLE15-176_55.fcs
Event Count: 3603

T cells
NLE15-176_55.fcs
Event Count: 3443

MOUSE 56

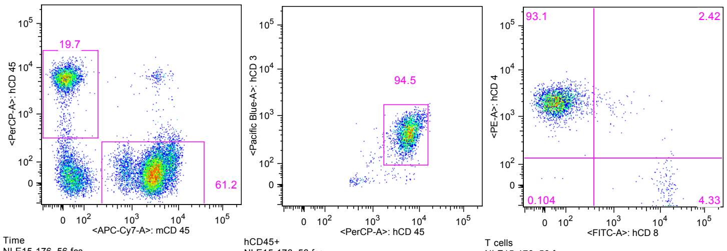

Time
NLE15-176_56.fcs
Event Count: 15500

hCD45+
NLE15-176_56.fcs
Event Count: 3055

T cells
NLE15-176_56.fcs
Event Count: 2887

MOUSE 57

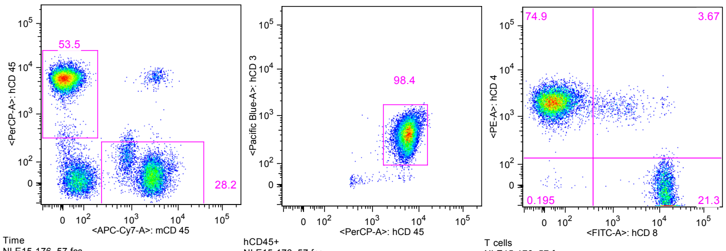

Time
NLE15-176_57.fcs
Event Count: 24326

hCD45+
NLE15-176_57.fcs
Event Count: 13003

T cells
NLE15-176_57.fcs
Event Count: 12794
